# Third‐order self‐embedded vocal motifs in wild orangutans, and the selective evolution of recursion

**DOI:** 10.1111/nyas.15373

**Published:** 2025-05-16

**Authors:** Chiara De Gregorio, Marco Gamba, Adriano R. Lameira

**Affiliations:** ^1^ ApeTank, Department of Psychology University of Warwick Coventry UK; ^2^ Department of Life Sciences and Systems Biology University of Torino Torino Italy

**Keywords:** great apes, isochrony, recursion, rhythm, tempo, vocal communication

## Abstract

Recursion, the neuro‐computational operation of nesting a signal or pattern within itself, lies at the structural basis of language. Classically considered absent in the vocal repertoires of nonhuman animals, whether recursion evolved step‐by‐step or saltationally in humans is among the most fervent debates in cognitive science since Chomsky's seminal work on syntax in the 1950s. The recent discovery of self‐embedded vocal motifs in wild (nonhuman) great apes—Bornean male orangutans’ long calls—lends initial but important support to the notion that recursion, or at least temporal recursion, is not uniquely human among hominids and that its evolution was based on shared ancestry. Building on these findings, we test four necessary predictions for a gradual evolutionary scenario in wild Sumatran female orangutans’ alarm calls, the longest known combinations of consonant‐like and vowel‐like calls among great apes (excepting humans). From the data, we propose third‐order self‐embedded isochrony: three hierarchical levels of nested isochronous combinatoric units, with each level exhibiting unique variation dynamics and information content relative to context. Our findings confirm that recursive operations underpin great ape call combinatorics, operations that likely evolved gradually in the human lineage as vocal sequences became longer and more intricate.

## Graphical abstract

Recursion, a core feature of language, was long considered unique to humans. We investigate rhythmic recursion in great ape vocalizations, analyzing Sumatran orangutan alarm calls and uncovering third‐order self‐embedded isochrony, with each level exhibiting unique variation dynamics and information content relative to context. Our findings suggest that recursive rhythmic structures underpin great ape call combinatorics and likely evolved gradually in the human lineage as vocal sequences became more complex.

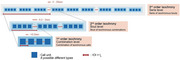

## INTRODUCTION

Language defines being human, but its evolution defies scientific explanation. Features once believed to be unique to language have been found to be shared with animal communication,^1^, ^2^, ^3^ helping to begin reconstructing the system that, among hominids, evolved to become language. However, virtually nothing is known about the evolutionary origins of the core operation that has been classically proposed to set language fundamentally apart from other animal systems^4^—*recursion*. Recursion is the capacity to embed structures within similar structures, creating hierarchical strata of elements/patterns nested within self‐similar elements/patterns.^5^, ^6^ Such operation enables the “infinite use of finite means,” making language open‐ended and generative.^4^ ^,^ ^7^ Everyday examples of recursion include Russian dolls nested inside other Russian dolls, computer folders filed inside other computer folders, the spirals of Romanesco broccoli organized into a spiral, trees, rivers, and arteries branching out into smaller branches, streams, and arterioles that themselves branch out. Recursion is ubiquitous in nature but seemingly absent in animal communication systems, making its evolutionary origin as language's core feature all the more enigmatic.

To assess the recursive capacities of (nonhuman) animals, artificial grammar experiments using humanmade tokens and rules have shown that other species can learn to perceive and process recursive structures after receiving dedicated training^8^, ^9^, ^10^, ^11^, ^12^ (but see Ref. 13). However, these studies have focused on taxa with long independent evolutionary histories from humans (e.g., birds, monkeys), and used stimuli that are alien to the species through language‐able intermediators (i.e., humans). These conditions neither represent realistic natural conditions before the emergence of language in the recent human lineage nor can they inform whether or how ancestral hominids *produced* and assembled recursive structures in the first place, leaving the origins of recursion virtually intractable. The seeming absence of evidence for recursive production capacities in animals, namely (nonhuman) great apes, which could reveal potential homologous, transient, or precursor stages for the evolution of recursion, has led some scholars to propose that recursion did not emerge as the result of a gradual step‐by‐step selective process, but instead, of a sudden all‐or‐nothing mutational event.^14^, ^15^, ^16^ Which of these views presents the most likely path for the evolution of language represents one of the most hotly debated issues in science since the mid‐20th century^17^, ^18^, ^19^.

**FIGURE 1:**
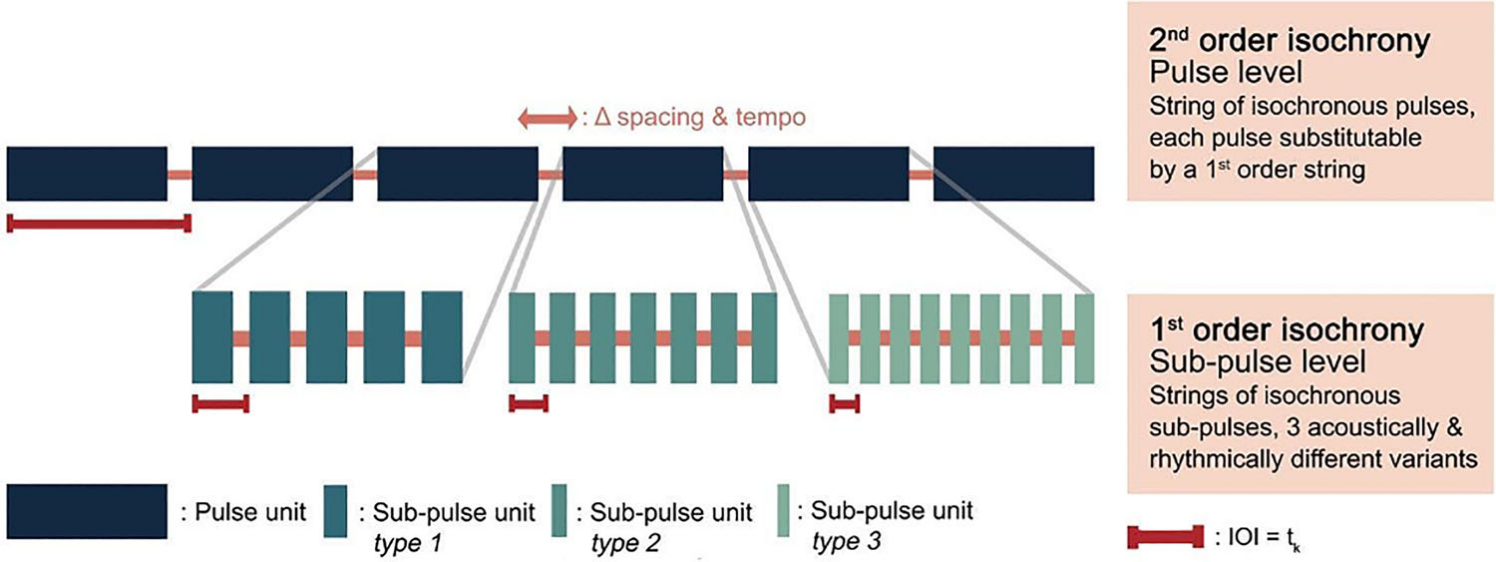
Recursive self‐embedded isochrony in Bornean male orangutan loud calls, as originally described by Lameira et al.^6^ The findings represent a case of second‐order self‐embedded isochrony underpinned by applying a recursive operation once.

Recently, the first evidence for recursive operations has, however, emerged in great ape call combinations, suggesting that these operations may have been hiding in plain sight. Lameira and colleagues^6^ analyzed the long calls of wild Bornean flanged male orangutans (*Pongo pygmaeus wurmbii*) produced as advertisement calls to attract females and deter other males—the most well‐studied orangutan vocal behavior^20^, ^21^, ^22^, ^23^, ^24^, ^25^—and detected that call units were organized in self‐embedded motifs along two different structural strata (Figure 1). At one level, call units, “pulses”, were organized in an isochronous fashion, that is, laid along a regular tempo with constant intervals separating each pulse from the next. Pulses could, however, subdivide into sub‐units, “sub‐pulses”, that were themselves organized in an isochronous fashion, showing regular tempo and constant intervals separating each sub‐pulse from the next. Remarkably, pulses could be categorized in three different types of sub‐pulses, all of them isochronous, but with different tempos and acoustically distinct from each other (Figure 1). These recursive motifs of isochrony within isochrony were unlikely to be byproducts of anatomical constraints or mere production artifacts (see Ref. 26), suggesting their production involved some degree of neuro‐motor processing to regulate and adjust vocal rhythmic structures across scales.

**FIGURE 2:**
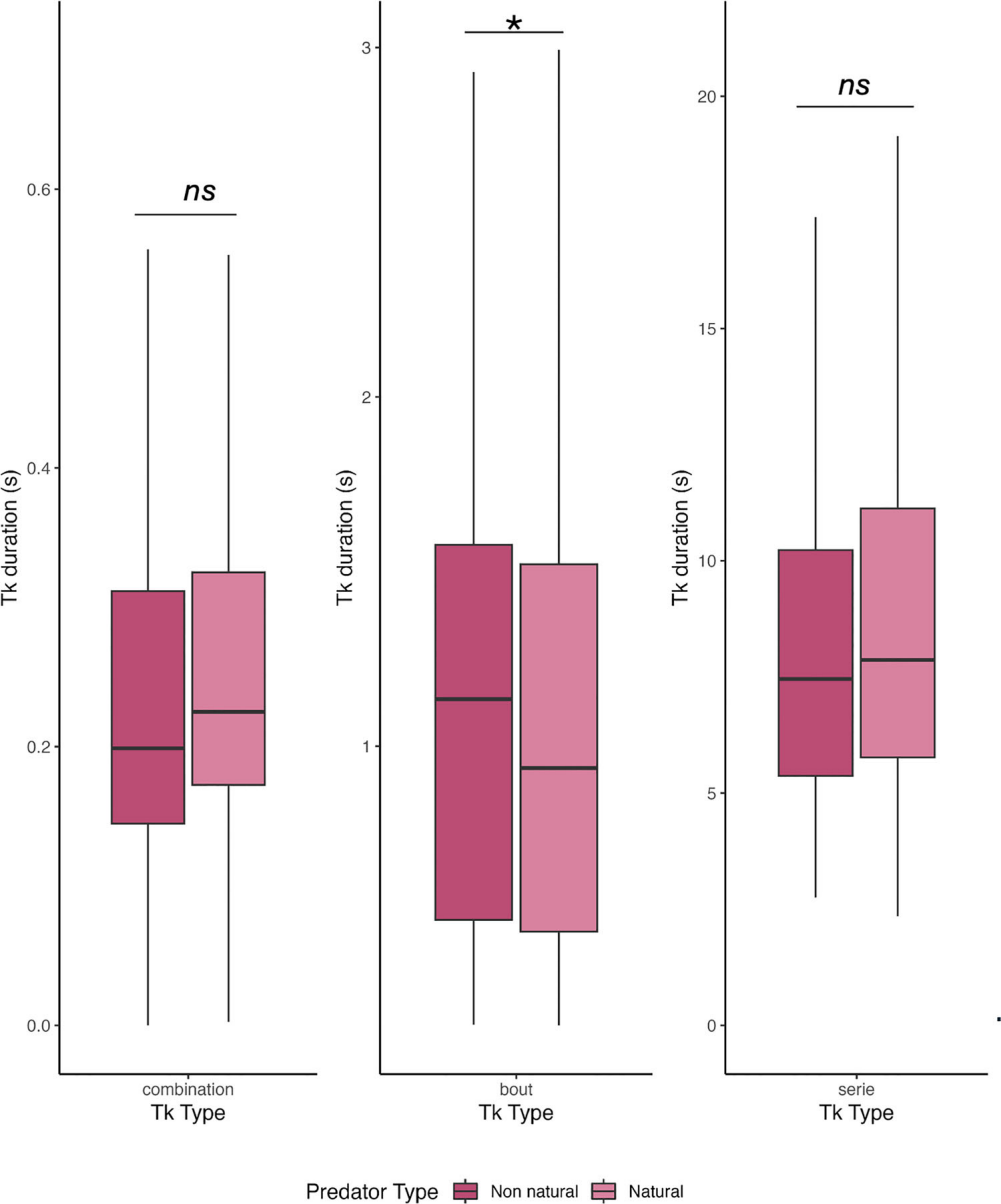
Tempo differences across levels for alarm calls given in response to natural (light pink) and non‐natural (pink) predators. From left to right: tempo of combinations, bouts, and series of alarm calls. * Denotes *p*‐value <0.05; ns denotes the absence of statistical significance.

The findings by Lameira et al.^6^ show that recursive operations can occur within the family Hominidae in the absence of full‐fledged language, indicating that homologous operations could have been in use by ancestral hominids. According to the principles of evolution by natural selection, such operations could have provided neuro‐computational and behavioral feedstock for the evolution of recursion in language through descent with modification based on shared hominid ancestry. These findings offer the first empirical comparative evidence supporting a step‐by‐step evolutionary scenario for the emergence of recursion along the human lineage, hinting at a possible resolution of the gradual versus saltationist debate Figure 2.

### Data‐driven hypothesis‐testing: Predictions for a gradual evolutionary scenario

If recursive operations were indeed available in the vocal toolkit of ancestral hominids and were evolutionarily relevant for the emergence of language, then four obligatory key predictions would need to be fulfilled. First, recursive operations among nonhuman hominids should not be restricted to one species or one location. Only then would recursive operations be sufficiently resilient to stochastic demographic variations, ecologic vagaries, and/or extinction events,^27^ especially in light of severe reductions in species richness suffered by the family Hominidae in the wake of paleoclimate change, starting 17 Mya (reviewed in Ref. 28). Taxonomic and geographic spread over extended periods of time would have provided the best conditions for selective processes to operate.

Second, recursive operations should not be restricted to one sex, given that ultimately any individual ought to be able of deploying recursion.

Third, recursive operations should not be restricted to one vocal behavior. Notably, to support the view that the use of recursive operations in ancient hominids would have been pertinent for language evolution, they ought to underlie different types of consonant‐like and vowel‐like calls, and combinations thereof, given that these eventually became the two elemental building blocks of the primary medium of language.^29^, ^30^, ^31^, ^32^, ^33^

Fourth, recursive operations should not be restricted to one context. They ought to occur across, be affected by, and help predict, different external objects or events. Recursive operations across contexts would be imperative for functional (i.e., informative) use, given that only then could recursive operations begin to offer fitness benefits, setting selective forces in motion. Environmental responsiveness and context‐sensitivity would, thus, be a requirement if recursive operations were to ultimately operate on elements encoding semantic information (e.g., words).

Note that these conditions, while essential for a gradual evolutionary pathway, inherently counter possible saltationist arguments. A single mutational event capable of engendering a major transformation in an ancestral hominid would be likely confined to a single species, location, vocal behavior, or context (had individuals hypothetically remained reproductively unaffected after such an event). Such a limitation would constrain mutation persistence, spread, and fixation within and across populations.

Given that initial proof for the presence of recursive operations exists for Bornean male orangutans’ long calls used for sexual advertisement,^6^ here we test the four above predictions by expanding orangutan call combinatorics research and examining the possible presence of recursive motifs in another great ape species, in a distinct island, and in the opposite sex, involving different call types and in a separate context, namely, in wild Sumatran females’ (*Pongo abelii*) alarm call responses in predation situations, which also represent the longest known strings of consonant‐like^32^ and vowel‐like^34^, ^35^ call combinations among hominids outside humans and language.

## METHODS

### Subjects and data collection

We tested seven wild adult female orangutans, habituated to human presence, who represented all adult female local residents of the Ketambe forest (3°41′N, 97°39′E) in Aceh, Sumatra, Indonesia, between 2010 and 2011, comprising about 450 hectares of lowland and hill dipterocarp rainforest. Subjects were presented with four predator models, consisting of a human experimenter walking on all fours on the forest ground draped over a sheet with one of four different types of print: tiger patterned (orangutans’ natural predator in Sumatra^23^), and three different prints simulating non‐natural predators: a multi‐colored abstract pattern, a white pattern with multi‐colored spots, and a plain white pattern. Once a subject gained sight of the model, the experimenter moved out of sight after 2 min. All alarm call responses (*n* = 14) were recorded using Marantz Recorder PMD‐660 with a Rode NTG2 Microphone between 7 and 20 m away from focal subjects. Responses in canopy ascent and stress physiological responses by the focals after sighting the predator model have been described in Lameira and Call.^2^

### Data preprocessing

Spectrograms were visually inspected in Raven Pro^36^ (version 1.6; window type: Hann; 3 dB filter bandwidth: 124 Hz; grid frequency resolution: 2.69 Hz; grid time resolution: 256 samples). Each call's starting point and duration were annotated by manually drawing a selection box around each call.

To evaluate the rhythmic structure of orangutan alarm calls, we considered three levels of analysis: (1) combination level: calls separated by 0.2 s or less were considered to represent a combination; (2) bout level: combinations separated by more than 0.2 and less than 2 s were considered to represent a bout; (3) series level: bouts separated by more than 2 and less than 20 s were considered to represent a series. When a silent gap between two calls was over 20 s, calls were considered to belong to different series. From 14 recordings, we obtained 48 different series.

For each level of analysis, we calculated the inter‐onset interval duration (aka: IOI, hereafter *t*_k_).^37^ For the combination/bout/series level, *t*_k_ corresponded to the time between the onset of a call/combination/bout and that of the following one, respectively. We obtained 2427 *t*_k_ values at the combination level, 454 *t*_k_ values at the bout level and 946 *t*_k_ values at the series level. We then calculated the ratio of two subsequent *t*_k_ (hereafter *r*_k_) for each level. This operation involved dividing the duration of a *t*_k_ by the sum of its duration and the duration of the following *t*_k_.^38^ We obtained 1319 *r*_k_ values at the combination level, 277 *r*_k_ values at the bout level, and 872 *r*_k_ values at the series level.

To investigate the occurrence of rhythmic categories, we divided the ratio distribution into on‐integer and off‐integer ratio ranges, centering the on‐integer ratio range around 1:1 (or 0.50—representing isochrony), 1:2 (or 0.33), 1:3 (or 0.25), 2:1 (or 0.66). Following previous studies,^37^, ^38^, ^39^, ^40^ a 1:1 on‐integer ratio was considered when *r*_k_ fell within the values between 0.444 and 0.555, and an off‐integer ratio when *r*_k_ fell between 0.4−0.444 and 0.555−0.6. The range of a 1:2 on‐integer ratio was considered *r*_k_ to fall within the values 0.308 and 0.364, and the off‐integer ratio to fall within the values 0.286−0.308 and 0.364−0.4.

The range of a 1:3 integer ratio was considered when *r*_k_ fell between 0.235 and 0.267, and an off‐integer ratio between 0.222−0.235 and 0.267−0.286. For the 2:1 ratio, we considered an on‐integer ratio from 0.636 to 0.692 and an off‐integer ratio from 0.6 to 0.636 and 0.692 to 0.714. We then counted all ratios situated within on‐ and off‐integer intervals for each level to statistically test the presence of rhythmic categories.

### Data analyses

#### Statistical model overview

We used nine generalized linear mixed models (GLMMs) using the *glmmTMB* package^41^ in R^42^ to investigate the distribution of *r*_k_ and one linear mixed model (LLM) using the *lmer* function^43^ to investigate the distribution of *t*_k_ at different hierarchical levels in orangutan alarm calls. The syntax and details for each model are specified in the following paragraphs. Before creating the models, we checked the distribution of each response variable with the package *fitdistrplus*,^44^ and residual distribution and model dispersion with the *Dharma* package.^45^ GLMMs were fitted using a Poisson distribution (count variable), and we set *ziformula* = 1, to account for the presence of zeros in the datasets. We also included an offset variable weighting the *r*_k_ count based on the width of the bin in the probability density curve. The LMM was fitted with a log‐transformed *t*_k_ duration to match a normal distribution. For all the models, we adopted a full versus null approach to test for the significance of our full models, comparing each model with the same one containing random factors only (Anova with chi‐sq argument).^46^ If null and full significantly differed, then the fixed factors affected the distribution of the response variable, and, therefore, we then applied post hoc tests (*emmeans*)^47^ to perform all pairwise comparisons for the levels of the fixed factors.

#### Tempo variation per context

To examine the potential influence of context on each level's tempo, we used an LMM per level to assess differences in *t*_k_ duration across predator models, treating predator models as belonging to one of two categories (natural vs. non‐natural) to assure sufficient statistical power. Per model, we log‐transformed *t*_k_ duration and used it as a response variable. Before running the models, we checked the distribution of the response variable, residual distribution, and model dispersion (as above). We used the interaction between the type of *t*_k_ (belonging to combination, bouts, or series) and the type of predator (natural, non‐natural). Individual identity was used as a random factor. The full model significantly differed from the null model, containing only random factors (χ^2^ = 8446.466, df = 5, *p* < 0.001). We then used a post hoc test to perform all pairwise comparisons between the types of *t*_k_ and the interaction *t*_k_ type and predator type.

#### Investigating rhythmic patterns within layers

For the combination level, we ran a model using as a response variable the *r*_k_ count of the intervals between different alarm calls in the same combination, and the *r*_k_ bin type as a fixed factor (factor levels: 1:1 off, 1:1 on). We used the file ID from which the ratios were extracted as a random factor. The comparison with the null model was significant (χ^2^ = 591.6478, df = 7, *p* < 0.001).

We created two subsets of *r*_k_ data, selecting only those calculated from alarms given in response to the natural predator model (i.e., tiger—subset 1) and those in response to non‐natural predators (i.e., pattern, spots, and white—subset 2), respectively. Each subset of *r*_k_ was used as the response variable, and the *r*_k_ bin type was used as a fixed factor (factor levels: off1:1, on1:1). We used the file ID from which the ratios were extracted as a random factor. The comparison with the null model was significant both for the model ran on the tiger dataset (χ^2^ = 244.5079, df = 7, *p* < 0.001) and the one ran on the non‐natural predator dataset (χ^2^ = 86.24725, df = 7, *p* < 0.001).

For the bout level, we used as a response variable the *r*_k_ count of the intervals between different alarm call bouts and the *r*_k_ bin type as a fixed factor (factor levels: 1:1 off, 1:1 on, 1:3 off, 1:3 on). We used the file ID from which the ratios were extracted as a random factor. The comparison with the null model was significant (χ^2^ = 278.9431, df = 7, *p* < 0.001).

We then created two subsets of data. In the first case, selecting only the *r*_k_ calculated from alarms given in response to the simulation of the presence of a natural predator (in this case, a tiger). In the second case, the *r*_k_ calculated from alarms given in response to the simulation of the presence of non‐natural predators (pattern, spots, and white). We used both datasets within the same model as the overall dataset. The comparison with the null model was significant both for the model ran on the tiger dataset (χ^2^ = 166.8937, df = 7, *p* < 0.001) and the one ran on the non‐natural predator dataset (χ^2^ = 166.897, df = 7, *p* < 0.001).

For the series level, we used as a response variable the *r*_k_ count of the intervals between different alarm call bouts and the *r*_k_ bin type as a fixed factor (factor levels: 1:1 off, 1:1 on). We also included an offset variable weighting the *r*_k_ count based on the width of the bin on the probability density curve. We used the file ID from which the ratios were extracted as a random factor. The comparison with the null model was significant (χ^2^ = 1413.258, df = 5, *p* < 0.001).

We then created two subsets of data. In the first case, selecting only the *r*_k_ calculated from alarms given in response to the simulation of the presence of a natural predator (a tiger). In the second case, the *r*_k_ calculated from alarms given in response to the simulation of the presence of non‐natural predators (pattern, spots, and white). We used both datasets within the same model as the overall dataset. The comparison with the null model was significant both for the model ran on the tiger dataset (χ^2^ = 149.9398, df = 7, *p* < 0.001) and the one ran on the non‐natural predator dataset (χ^2^ = 81.94174, df = 7, *p* < 0.001).

## RESULTS

### Tempo variations per context

**FIGURE 3:**
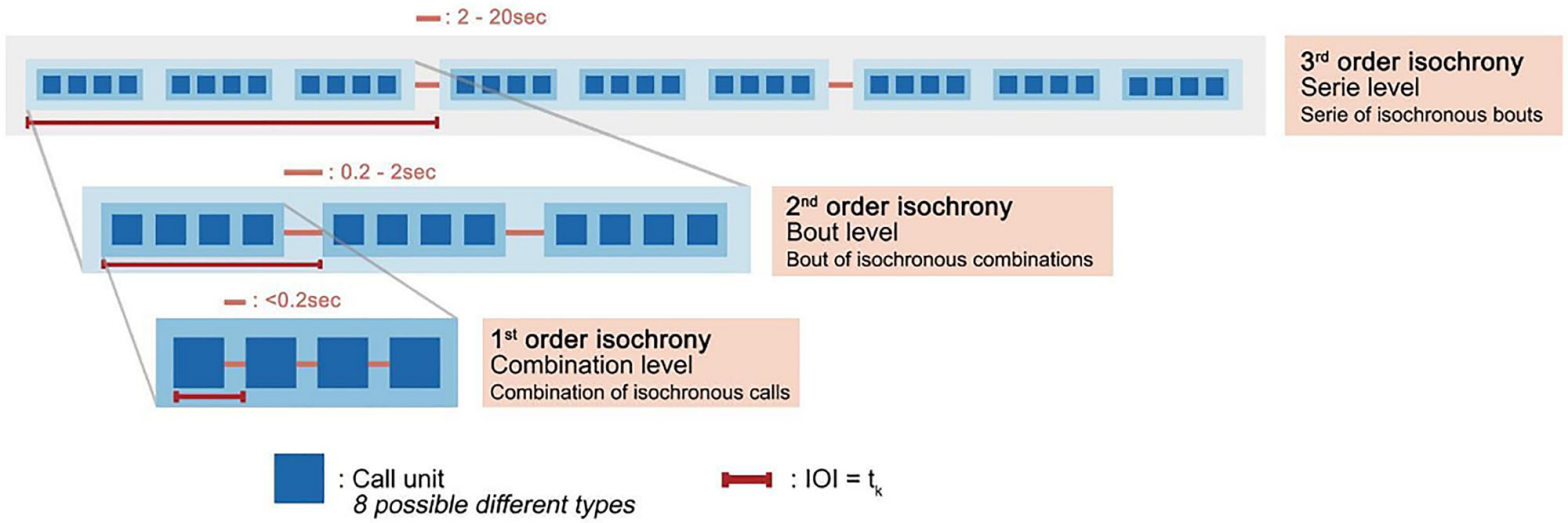
Recursive self‐embedded isochrony in Sumatran female alarm calls. Findings represent a case of third‐order self‐embedded isochrony underpinned by applying a recursive operation twice. Dark blue blocks represent call units of eight different possible call types. Light‐blue blocks containing dark squares represent bouts. Light gray blocks containing light‐blue blocks represent series. Orange lines between blocks represent silent intervals. Red lines represent inter‐onset *t*_k_ intervals.

The average *t*_k_ of alarm combinations was 0.259 ± 0.138, for bouts was 1.11 ± 0.678, and for series was 8.74 ± 4.18. The ratio between *t*_k_ of different layers was approx. 2:9:67. We found that the tempo of a series of bouts given in response to a non‐natural predator was slower than the one in response to natural predators (non‐natural bouts vs. natural bouts: estimate = 0.1669, SE = 0.0553, df = 3115, *t*‐ratio = 3.017, *p* = 0.031). At the same time, no differences were detected between other levels (non‐natural combinations vs. natural combinations: estimate = −0.0367, SE = 0.0373, df = 1070, *t*‐ratio = −9.983, *p* = 0.923; non‐natural series vs. natural series: estimate = −0.0310, SE = 0.00463, df = 2041, *t*‐ratio = −0.671, *p* = 0.9851; Figure 3).

**FIGURE 4:**
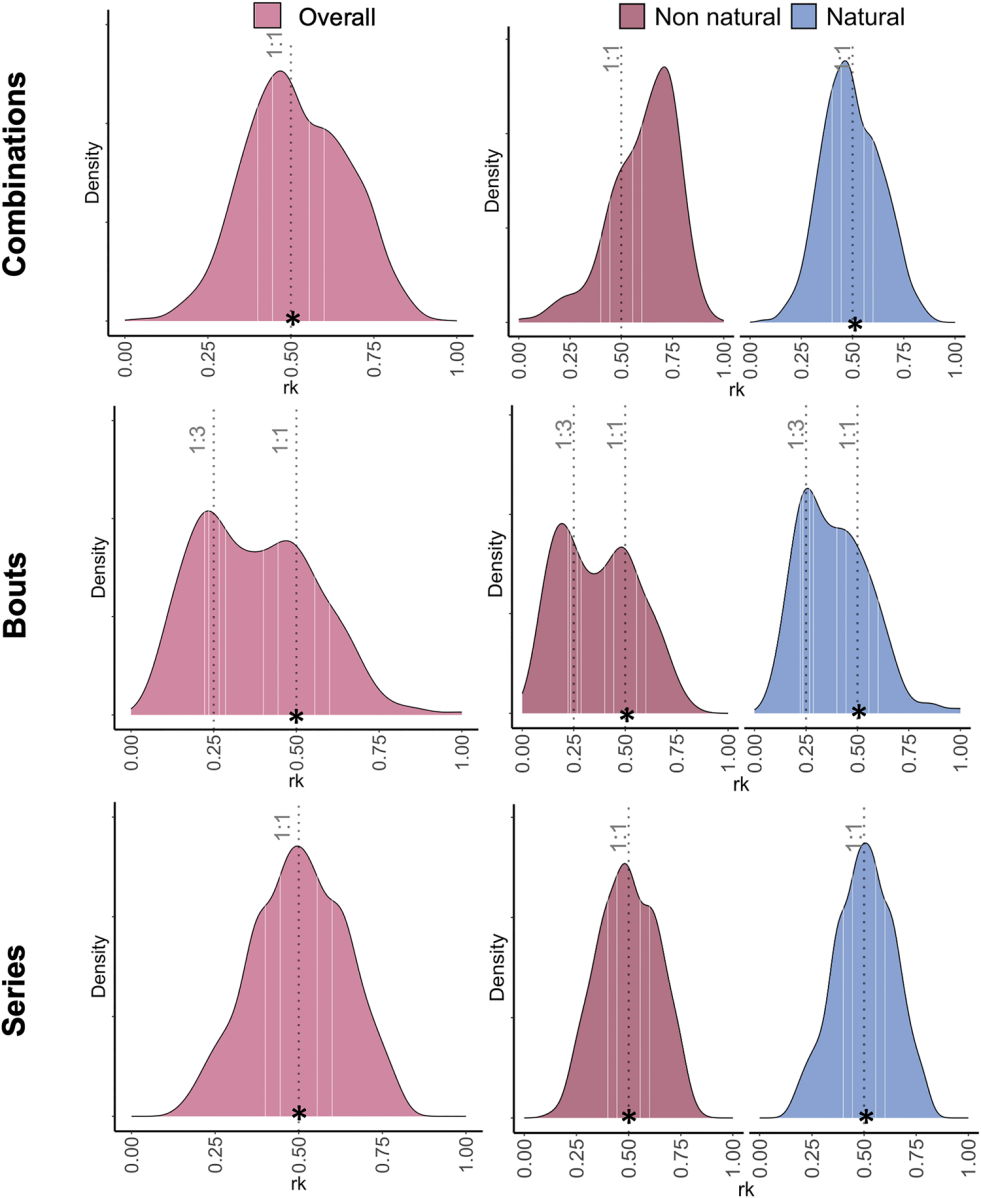
*r*_k_ distributions for alarm calls in the three layers. From top to bottom: probability density functions of *r*_k_ for the combination level, overall and based on predator type; probability density functions of *r*_k_ for the bout level, overall and based on predator type; density functions of *r*_k_ for the series level, overall and based on predator type. * Denotes *p*<0.05; statistically significant matching between the empirical distribution and a small integer ratio rhythmic category.

### Isochrony across levels

The visual inspection of the density distribution suggested the presence of a 1:1 ratio (isochrony) for the combination level (Figure 3A), bout level (Figure 3B), and the series level (Figure 3C). Additionally, the bout level exhibited a bimodal density distribution, with a second peak around 1:3. Hence, we also tested for the presence of a 1:3 categorical rhythm in the bout level Figure 4.

### Isochrony at the combination level

We found that the temporal organization of alarm calls within a combination was isochronous (1:1 off vs. 1:1 on; estimate = −0.345, SE = 0.108, z‐ratio = −3.186, *p* = 0.031). The same was true also for calls emitted in response to natural predators (1:1 off vs. 1:1 on; estimate = −0.352, SE = 0.111, z‐ratio = −3.165, *p* = 0.001), but not the case for calls emitted in response to non‐natural predators (1:1 off vs. 1:1 on; estimate = −0.474, SE = 0.449, z‐ratio = −1.056, *p* = 0.291).

### Isochrony at the bout level

The temporal organization of consecutive bouts of alarm calls was overall isochronous (1:1 off vs. 1:1 on; estimate = −0.816, SE = 0.227, z‐ratio = −3.599, *p* < 0.001). We obtained the same result and also when considering the type of predator: bouts of isochronous combinations were isochronous when given in responses of natural (1:1 off vs. 1:1 on; estimate = −0.655, SE = 0.282, z‐ratio = −2.322, *p* = 0.020) and non‐natural predators (1:1 off vs. 1:1 on; estimate = −1.09, SE = 0.385, z‐ratio = −2.825, *p* = 0.005).

The density distribution showed a bimodal distribution (Figure 3), hence, we also investigated the presence of an additional rhythmic category. Because the second distribution peak occurred at *r*_k_ = 0.25, we specifically investigated the potential presence of the corresponding 1:3 rhythmic category. We found that the ratio values did not match precisely the 1:3 on‐integer (1:3 off vs. 1:3 on; estimate = 0.0488, SE = 0.312, z‐ratio = 0.156, *p* = 0.876). This was also true when considering predator type: bouts of isochronous combination did not match the 1:3 integer ratio in response to natural (1:3 off vs. 1:3 on; estimate = −5.66e‐10, SE = 0.378, z‐ratio = 0.000, *p* = 1.000) and non‐natural predators (1:3 off vs. 1:3 on, estimate: = 0.154, SE = 0.556, z‐ratio = 0.277, *p* = 0.782).

### Isochrony at the series level

We found that consecutive bouts of alarm calls were isochronous (1:1 on vs. 1:1 off; estimate = 0.429, SE = 0.156, z‐ratio = 2.753, *p* = 0.006). The same was true also when considering a series of alarms given in response to natural predators only (1:1 off vs. 1:1 on; estimate = −0.546, SE = 0.122, z‐ratio = −4.467, *p* <0.001) and non‐natural predators (1:1 off vs. 1:1 on; estimate = −0.701, SE = 0.193, z‐ratio = −3.626, *p* < 0.001).

## DISCUSSION

Our results demonstrate the presence of recursive self‐embedded vocal motifs organized across three hierarchical strata in the alarm calls of female Sumatran orangutans. These results expand on previous findings in the long calls of male Bornean orangutans, in direct agreement with the predictions defined at the start of the study for the gradual evolution of recursion. Together, these two converging lines of evidence show that recursive vocal operations were likely present in ancient hominids across taxa and geography, likely deployed by males and females, and dynamically used to encode information about external objects and events in various call types. These conditions would have been necessary and sufficient to allow natural selection processes to target recursive vocal combinatorics based on their proximate functions, information content, and communication benefits.

Our findings provide, to our knowledge, the first empirical support to the view that ever more powerful recursive capacities could have been selected for and evolved incrementally. In one context, individuals invoked a recursive^48^ operation once, generating a second‐order self‐embedded motif in the form of [isochrony^A^ [isochrony^a,b,c^]],^6^ while in another context, individuals invoked a recursive operation twice, generating a third‐order self‐embedded motif in the form of [isochrony [isochrony [isochrony]]] (or isochrony^3). To add new stratifying levels of self‐nested isochrony (isochrony^{n}), the limit seems to be imposed by the length of the alarm call responses. This is because each new level of isochrony requires a considerable increase in the time scale of the response. For example, it is possible that female orangutan alarm call series also organize themselves isochronally, generating a fourth‐order motif. However, too few responses in our sample were sufficiently long to allow statistical testing of this possibility. This may be something that future predator model experiments with wild orangutans will help reveal.

### Layers of information created by recursive operations

Our results revealed differences in temporal organization across levels in natural versus non‐natural predator models. Notably, the predator model type affected a combination isochrony and bout tempo. At the bout level, this result is in line with other instances in the animal world where alarm calls^49^, ^50^, ^51^ and alarm call rhythm^40^, ^48^ convey information about external threats and predators and when certain threats are perceived as posing higher levels of danger and urgency than others. The encoding of predator information via isochrony versus nonisochrony has, however, thus far, not been reported in other species, to the best of our knowledge. Orangutan alarm calls show that individuals segregate predator information and encode that information differently across different structural levels. Traditionally, animal call sequences are assumed to be assembled in a linear and memoryless fashion, and therefore, to exhibit one sole structural layer.^52^ A purely linear assembly process may be insufficient, however, to explain the combinatoric complexity of several call sequences across taxa.^53^ Further investigation into the possible presence of vocal structural strata in phylogenetically distance species, as identified here in orangutans, may allow comparing for the first time how different taxa may allocate (codes of) information across different structural layers. For example, in humans, speech isochrony has been shown to improve intelligibility across languages of different rhythm classes, providing an optimal structural template for predictable information encoding.^54^ Comparative research across taxa and possible experimental work with orangutans in the wild could help explain why predator information is encoded as on‐ or off‐isochrony at the combination level but via different isochronous tempos at the bout level.

Both instances of predator information encoding based on on/off‐isochrony and variation in isochrony tempo support the view that this recursive system has evolved for, and facilitates, effective communication, which ought to be expected to be at a selective premium among dispersed social organizations, as is characteristic for wild orangutans.^55^, ^56^

### Isochrony at larger time scales

Our findings reveal for the first time, to our knowledge, the presence of an isochronous temporal organization at a large timescale, a regular tempo with >10 s between each sound onset. Previous work on primate vocal rhythms has focused primarily on temporal intervals with durations of <5 seconds.^6^, ^37^, ^39^, ^40^, ^57^ Five seconds is also hypothesized to represent an upper limit for meter perception and synchronization in humans.^58^, ^59^, ^60^ This suggests that producing rhythmic sounds separated by longer intervals is probably challenging for producers and receivers. What could then explain why orangutans produce alarm calls with such extended regular intervals?

Our study was purely observational, but we can offer two possible proximate reasons. Using recurring silence gaps that are at least 10 s long during an alarm call response is likely to allow individuals to systematically monitor and gather information from their environment, being it about the presence or movement of the predator they have just encountered or possible nearby conspecifics. Using isochrony at this scale would then allow individuals to adjust their ensuing antipredator and general behaviors accordingly, something difficult to achieve with recurring gaps shorter by one or two orders of magnitude (i.e., at first‐ and second‐order isochrony). Second, third‐order isochrony with >10 s gaps could also represent a byproduct of optimal resting times between series of alarm calls.

There are multiple examples of patterns in music with intervals equally as lengthy as orangutans’ alarm call series, including Bolero's ostinatos, “cycles” in African and Indian music, isorhythm in medieval music, rondo in classical music, and passacaglia and chaconne in Baroque music. Because rhythm tempos progress in these instances over cycles of >10 s, they can only emerge and be detected as extended compositions that surpass the length of typical ordinary speech exchanges and typical laboratory stimuli. This could begin to explain why this temporal scale of organization has not, and cannot often be, investigated. Our results invite a “zoom out” of the empirical window for the study of acoustic communication and vocal rhythms in long sequences across taxa, including humans (e.g., speeches). Nonetheless, the parallel with music is limited. Long‐cycle musical motifs unfold over several bars and (therein) beats, thus, the ratios between these levels must be necessarily even integers. This differs from our observation in wild orangutans, where the three layers of isochrony were related by the odd ratio 2:9:67.

### Is vocal output underpinned by recursive neural architecture

Encoding information in the temporal structure across hierarchical layers supports the view that, once present, recursive assembly operations could provide proximate benefits, multiplying the amount of information that a message may encode and diversifying how the same message can be encoded. However, most signal systems in nature are, and have been, also shaped by similar benefits and selective forces, and yet, the emergence of different hierarchical layers has not ensued in most cases. Why recursive operations have only been detected in a hominid remains, thus, unclear. A possible answer could rest on the size and scale of the hominid brain. Human^61^, ^62^, ^63^ and primate brains^64^ are organized in layers of recursive self‐embedded structures that have functional consequences.^65^, ^66^ The discovery of second‐order^6^ and third‐order self‐embedded vocal motifs in orangutans (this study) raises the question of whether recursion might represent an emergent neural network effect. Once a certain threshold of neural complexity is reached, a brain that is recursively organized may inherently produce vocal output that is also recursively organized, especially when involving long and complex structures composed of different types of units that encode different types of information, and thus, recruit various neural populations and brain regions. Indeed, language is underpinned by neural networks that differ in their functional time window, where interleaved networks operate at the scale of one, four, and six words,^67^ posing an intriguing parallel with our findings in how another hominid species also temporally organizes combinations of consonant‐like and vowel‐like calls.

Classically, primate vocal output is seen as a sonic cast of its vocal anatomy.^34^, ^68^ Our findings tentatively raise the possibility that the organization of primate vocal output is also, to some degree, a combinatoric cast of its underlying neural architecture. This possibility is consistent with new neurological evidence showing that language is a brain‐wide phenomenon that cannot be reduced to single circuitries and especially is based on recursive structural patterns of neural organizations.^65^, ^69^, ^70^, ^71^, ^72^, ^73^, ^74^, ^75^ The possibility that vocal recursion is an embodied feature of the primate brain^76^, ^77^, ^78^ may start to be tested through new comprehensive and detailed rhythmic and combinatoric analyses of long vocal sequences produced across taxa and branches of the primate order.

## AUTHOR CONTRIBUTIONS

C.D.G. and A.R.L.: Conceptualization. C.D.G.: Methodology. C.D.G.: Investigation. A.R.L. and M.G.: Supervision. C.D.G. and A.R.L.: Writing—original draft. C.D.G., A.R.L., and M.G.: Writing—review and editing. C.D.G. and A.R.L.: Visualization. All authors contributed to the article and approved the submitted version.

## CONFLICT OF INTEREST STATEMENT

The authors declare no competing interests.

## PEER REVIEW

The peer review history for this article is available at: https://publons.com/publon/10.1111/nyas.15373.

## Data Availability

The data that support the findings of this study are available from the corresponding author upon reasonable request.
